# Diagnosis and therapy of functional tremor a systematic review illustrated by a case report

**DOI:** 10.1186/s42466-020-00073-1

**Published:** 2020-12-03

**Authors:** Michael Bartl, Rebekka Kewitsch, Mark Hallett, Martin Tegenthoff, Walter Paulus

**Affiliations:** 1 Department of Clinical Neurophysiology, University Medical Center, Robert Koch Str. 40, 37075 Göttingen, Germany; 2 National Institute of Neurological Disorders and Stroke, National Institutes of Health, Building 10, Room 7D37 10 Center Drive, Bethesda, MD 20892-1428 USA; 3 Department of Neurology, BG-University Hospital Bergmannsheil Bochum, Ruhr-University, Bürkle de la Camp-Platz 1, Bochum, 44789 Germany

## Abstract

**Background:**

Diagnosis of functional movement disorders and specifically functional tremor (FT) (representing 50% of them) remains demanding. Additionally, due to heterogeneity of the disorders, structured concepts and guidelines for diagnosis and therapy are difficult to establish. Ascertaining the state of knowledge to derive instructions for operating procedures is the aim of this review.

**Main text:**

Based on a standardized systematic literature research using the term “psychogenic tremor” in the MEDLINE database dating back ten years, 76 studies were evaluated. Conventional features of FT are variability of frequency and amplitude. Further, response to distraction by motor and cognitive tasks is a key diagnostic feature in differentiation between organic and functional origin. A variety of electrophysiological tests have been evaluated including surface electromyography and accelerometry to establish laboratory-supported criteria for diagnosing tremor. Also, finger tapping tests have been used to identify FT, showing positive potential as supplementary evidence.

Imaging studies in general are mostly underpowered and imaging cannot be used on an individual basis. Therapeutic studies in FT often have a diagnostic component. Cognitive behavioral therapy should be the preferred psychological treatment independent of additional psychiatric symptoms. Other psychotherapeutic methods show lack of evidence concerning FT. Relaxation techniques and physiotherapy are an important additional feature, especially in children and adolescents. In regard to drug therapy, randomized and blinded trials are not available. A significant decrease in rating scales could be detected after active, not sham repetitive transcranial magnetic stimulation with a long-lasting effect. Also root magnetic stimulation seems to be effective. The clinical feature of tremor entrainment in FT can be used in combination with biofeedback as so-called tremor retrainment, using self-modulation of frequency and severity, to bring the movements under volitional control.

**Conclusion:**

Diagnosis and treatment of FT is challenging and should include a combination of intensive clinical examination and targeted addition of standardized testing, especially electrophysiological methods. Often therapeutic effects have a diagnostic component. A multimodal strategy, considering psychological factors as a potential origin as well as maintaining effects seems to be most effective.

## Introduction

The diagnosis of functional movement disorders (FMD, previously commonly referred to as psychogenic movement disorders) in general and specifically for tremor remains an ongoing clinical challenge. In individual patients it can be difficult clinically to clearly separate organic from functional movement disorders. Thus, there is a great demand for objective parameters allowing a better separation. On the other hand, this can be seen as a vicious circle as long as the clinical diagnosis remains the gold standard. Functional tremor (FT) is the most common form by representing 50% of functional movement disorders. According to the international classification of diseases in Version 10, the code F44.4 describes “psychogenic movement disorders (PMD)”, including functional forms of tremor.

Axis I in the new classification of tremor of the Movement Disorder Society is phenotype, and functional tremors are characterized by criteria such as inconsistency, sudden onset of symptoms, and variable features in terms of topographic distribution, frequency and activation characteristics [6]. A differentiation between involuntary tremor, like FT, and voluntary tremor, like malingering, is made in the classification; however, their differentiation remains difficult. Fortunately, malingering in most medical practice is uncommon.

Reduction of quality of life due to functional movement disorders, especially due to FT, is high. Structured data and concepts for diagnosis and therapy are difficult to establish due to heterogeneity. The aim here is to ascertain the current state of knowledge of the last 10 years and to derive instructions for operating procedures and future perspectives [26, 27].

## Methods

**Fig. 1:**
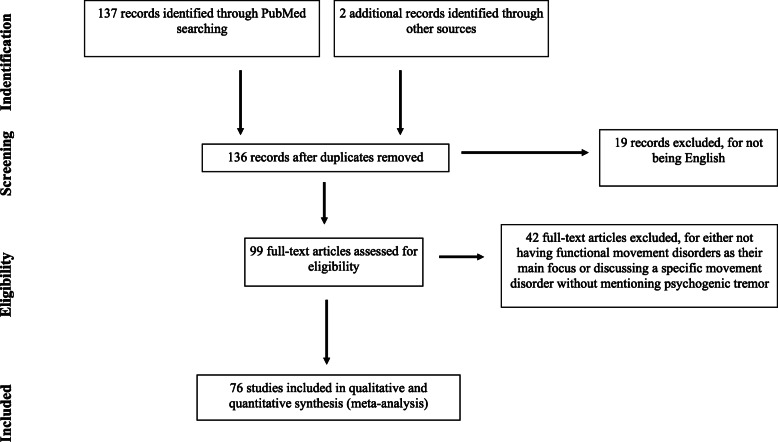
flow of information, systematic review

Based on a standardized procedure analogous to the PRISMA criteria for systematic literature research [28], a database analysis was performed. The National Library of Medicine’s MEDLINE database was searched for papers published in English, dating back ten years, using the term “psychogenic tremor” and reviewed here. A literature search with the preferred term “functional tremor” was also carried out. However, this turned out to be less effective due to the unspecific term “functional”. This resulted in a tremendously high number of hits, often without any content related to the topic. Studies that did not focus on psychogenic tremor or other functional movement disorders were excluded. Finally, 76 studies were categorized into the following categories: Diagnostics in functional tremor, therapy studies (both reported below), case reports, general overviews, Parkinson disease and other movement disorders (Additional file 9: Supplementary material). The detailed process of literature search and study selection can be seen in Fig. 1. Further, we categorized the studies into three different categories (low-, moderate- and high rated) according to their quality levels. The rating category of the study is marked behind the citation with the abbreviation LR/MR/HR. The assignments and the respective quality criteria are shown in Table 1.

**Table 1:** Study selection criteria

Study category	Quality criteria
Low rated (LR)	Case reports, reviews and clinical studies without standardized diagnostic criteria, evaluations scales and protocols
Moderate rated (MR)	Clinical trials with low patient numbers, lack of control groups, Reviews with incomplete study entry on validated basis
High rated (HR)	Controlled trials, reviews with standardized procedures, validated protocols and sufficient methodology

## Results

### Study categories

#### Diagnostics in functional tremor

Our systematic research identified 19 studies on diagnostic methods and their individual clinical value including 11 studies on patients (methods shown in Table 2). Conventional features (see Table 3) of functional tremor are variability of frequency and amplitude. The response to distraction is a key diagnostic feature in the differential diagnosis between organic and functional movement disorders. Distraction can be achieved by a variety of motor and cognitive tasks.

**Table 2:** Listing of diagnostic methods in functional tremor

diagnostic methods	definition	associated studies
**Test battery**	The EMG based tests: tapping performance, tapping response at different frequencies, testing of the ballistic movement response, tonic coactivation, coherence testing, loading tests have been shown as an effective tool to differentiate different tremor forms in a neurophysiological test battery	[27]
**Electromyography (EMG)**	EMG can help to differentiate between different tremor forms, by using surface EMG and time-frequency analysis. Variation in tremor frequency and between different muscles can be recorded. In functional tremor fewer limbs and limb segments are involved than in essential or Parkinson tremor. Further, the variability of the frequency is higher. The term “frequency dissociation” describes frequency differences higher than 0.1 Hz between two extremities. This can usually not be detected in functional tremor but in organic forms.	[20]
**somatosensory evoked potentials (SEP)**	During self-initiated movements a reduction in the SEP amplitude can be found at the onset of the movement. This is called sensory attenuation (SA) and is reduced in functional movement disorders	([20, 22])
**EMG coherence and cumulant analysis**	Recording EMG of muscle pairs and performing coherence and synchronicity analysis shows different patterns in postural upper limb tremor occurring in different tremor associated movement disorders	[32]
**Wavelet coherence analysis**	Method that can detect coherence variations and phase differences between EMG signals. This can help to differentiate between organic and functional tremor forms and is more precise than conventional coherence analysis.	[21]
**transcranial magnetic stimulation (TMS)**	In a study comparing organic and functional dystonia the motor cortical inhibition (short interval intracortical inhibition, SICI) measured by TMS showed abnormal values in both organic and non-organic groups. Further, paired associative stimulation (PAS) was abnormally high in the organic patient group and normal in the functional disorder group. They also found enhanced facilitation of the motor evoked potentials (MEP) in organic dystonia patients, but not in the functional controls. This could be validated in TMS recordings using cortical inhibition (SICI, long interval intracortical inhibition, LICI and cortical silent period, cSP). A reduced cortical inhibition is discussed as a precipitating factor for both organic and non-organic dystonia.	[20]
**pre-movement potential**	By using back averaging, in simultaneous recordings of the EMG and EEG it can be analysed if the EMG activity is preceded by EEG activity. This so-called movement related cortical potentials (MRCP) precede the onset of voluntary movement that are self-initiated. Two components of the Bereitschaftspotential (BP) and the reafferent potential recorded after the EMG activity can be differentiated. The involuntary movements in patients with FMD have a BP with appearance similar to that of normal voluntary movements.	[20]
**functional brain imaging (SPECT)**	Distinct patterns of cerebral perfusion at rest and during motor tasks could distinguish between functional and essential tremor from ET. A study showed a deactivation of the default mode network	[8]
**PET scan**	Possibly, abnormal sensory integration is a part of the pathogenesis of functional tremor. PET showed a hypermetabolism of the posterior parietal lobes bilaterally in patients	[19]
**Polymyography coupled to accelerometry**	Multiple recordings of muscle activity coupled with frequency analysis can be used to differentiate between different tremor forms in experimental study designs. They characterize the typical features like spontaneous variability of the tremor frequency and frequency entrainment induced by contralateral rhythmic tasks	[2]

**Table 3:** Clinical features of organic tremor evaluated and described in studies

**Clinical features**	
A typical feature of PMD is its paroxysmal nature and a **sudden onset** of the symptoms with no progression [16]	
The symptoms are (almost) maximal **at or near onset** [16]	
The tremor can have **different directions** and change them over time, this can especially be seen in mass loading testing ([2, 16, [20])	
Frequency analysis can help to differentiate between organic and functional tremors. Based on a classification of frequency into low (< 4 Hz), medium (4–7 Hz) and high (> 7 Hz) tremors with frequency > 11 is usually organic, but there is no rule with lower frequencies. Essential tremor is often 6 or 7 Hz, many tremor frequencies were suggested between **6 and 11 Hz to be often functional** ([1, 2, 16, 20])	
The amplitude, frequency as well as direction show a higher variability in functional tremor than in organic forms, usually the frequency of organic tremor does not show a variation higher than 0.5 to 1.0 Hz, in functional tremor a **larger variation in frequency** may be observed ([1, 2, 16, 20])	
Tremor shows different reactions to **distraction** and can cease or vary in intensity or frequency ([16, 20])	
In an **entrainment** test, the tremor takes up the frequency of a tapping in an unaffected extremity e.g. finger tapping ([16, 20])	
Often functional tremor can be precipitated by **suggestion** [16]	
Impaired walking and standing without a tendency to fall is often detected in functional tremor, meaning swaying gait and apparent **balance problems without falling** [16]	

To distinguish functional from organic origin in different movement disorders a variety of electrophysiological tests have been evaluated with the aim to provide objective criteria. Surface electromyography and accelerometry, pre-movement potentials electroencephalography, jerk locked back averaging, somatosensory evoked potentials and transcranial magnetic stimulation (TMS) were used to identify typical features of functional movement disorders like a higher variability and entrainability, coactivation, distractibility and increase in the amplitude and frequency on mass loading tests. Movement related cortical potentials such as a Bereitschaftspotential can be seen in functional myoclonus, but this assessment is not useful for functional tremor. Further, presence of triphasic muscle contractions and an absence of co-contraction suggests functional myoclonus [20] (HR).

An electrophysiological test battery to distinguish functional tremor and organic tremor with both high sensitivity (89.5%) and high specificity (95.5%) recorded EMG and accelerometry during a loading test, response to ballistic movements, a coherence test, tonic coactivation, tapping performance and tapping response. Thirty eight patients with functional tremor achieved higher scores on the test battery than the 73 patients with organic tremor so the authors proposed it as the basis of laboratory-supported criteria for diagnosing tremor. The scores only showed an overlap in the loading test [27] (HR).

The EMG recordings can be extended by using wavelet coherence analysis (WCA). Phase differences and coherence variations between EMG signals can be analyzed using the area under the receiver operating characteristic curve (AUC-ROC) against a standard coherence analysis. With higher WCA values a differentiation between organic and functional tremor was possible by analyzing patient groups with different tremor forms [21] (HR).

Recording muscle pairs (wrist and elbow extensors) with EMG and performing coherence and synchronicity measurements seems to be an additional tool in the assessment of postural upper limb tremor, showing a higher synchronicity pattern in Parkinson as well as functional tremor than in essential tremor forms [32] (HR).

Finger tapping tests (FTT) have been used to identify tremor with a functional cause. The subjects performed several 30 s trials using alternate hands. Afterwards an FTT mean score was calculated. When comparing 195 patients with various tremor forms with 130 healthy adults FTT scores were significantly lower in FT when compared to the other diagnostic groups. With a cutoff FTT ratio of 0.670 or less, testing was 89.1% specific and 76.9% sensitive for the diagnosis of FT. Authors concluded that this test can be used as supplementary evidence in the diagnosis of FT [7] (HR).

In 10 patients and 5 healthy controls functional brain imaging identified tremor as essential (ET) or functional (FT) with different distributions of cerebral blood flow between the groups to distinguish the tremor forms. SPECT imaging at rest and during a tremor inducing motor task was performed. In ET, rest imaging revealed increased rCBF (relative cerebral blood flow) in cerebellar hemi- spheres and left inferior frontal gyrus. During the motor task, ET patients demonstrated increased rCBF in the supplementary motor area (SMA) and contralateral motor cortex and reduced rCBF in the cerebellum and visual cortex. In contrast, In FT imaging at rest revealed an increased relative cerebral blood flow (rCBF) in the left inferior frontal gyrus as well as left insula. Imaging during motor task revealed increased rCBF in the cerebellum and reduced rCBF in the anterior regions of the default mode network [8] (HR).

Differences in the regional metabolic activity of the brain could be an option for differential diagnosis. In positron emission tomography (PET) hypermetabolism was seen symmetrically in both posterior medial lobes in FT, which was suggested to reflect abnormal activity with sensory integration [19] (LR). In general, most imaging studies are underpowered and thus may have many false positives. And, certainly imaging cannot be used on an individual basis.

Objective criteria useful for a positive diagnosis of functional movement disorders are highly recommended. A specific recording strategy should be applied. Polymyography coupled to accelerometry can be used to demonstrate the major electrophysiological criteria of FT, like spontaneous variability of tremor frequency and frequency entrainment during motor tasks of the contralateral side. Further, paradoxical increase of tremor amplitude during mass loading tests, a co-activation preceding the tremor onset, and an alteration of voluntary contralateral motor tasks are characteristics of FT [2] (HR).

It is suggested that functional movement disorders are more common in females. Addressing this question clinical and demographic features of males and females with FMD were evaluated [5] (HR). Of 196 FMD patients, only 30% were male. Further, they had an older age at disease onset as well as at the time of evaluation. Female patients were overrepresented in children and adolescents, but the genders were represented equally in patient older than 50 years.

#### Therapy studies

Therapeutic studies (overview Table 4) also have a diagnostic component. The more efficient a specific therapy is the more likely is the confirmation of the suspected diagnosis. In functional disorders the greater the therapeutic effort particularly in experimental approaches the larger an expected placebo effect will be. Placebo effects of course also occur in organic diseases. They are however expected to be larger in functional disorders. Finally, this will not significantly help in the diagnostic process.

**Table 4:** Therapeutic methods evaluated to treat functional tremor

therapeutic approach	type of application	associated studies
**repetitive transcranial magnetic stimulation (rTMS)**	Repeated low-frequency (0.25 Hz) magnetic simulation over the motor cortex or the spinal roots on the symptomatic side showed symptom improvement in functional tremor patients	([9, 13, 14, 31])
**tremor entrainment**	Tremor entrainment in form of tactile and auditory external cueing combined with real-time visual feedback as a short-term treatment can be effective in functional tremor therapy	[11]
**Pharmacological treatment**	drugs (e.g. propanolol, primidone, gabapentin, clonazepam, botulinum toxin, trihexyphenidyl) known from organic tremor treatment can be used with more or less success in FT therapy	([25, 33])
**psychodynamic psychotherapy (PDP)**	PDP can lead to an improvement in patients suffering from functional tremor. The prediction of responding rates is very challenging and prospective studies are generally lacking.	([18, 29])
**cognitive-behavioral therapy (CBT)**	CBT is an approach to instruct patients in identifying cognitive as well as physiologic responses experienced with stress. It aims to interrupt automatisms learned in association with the functional movement disorder. Even if the amount of data are small, single blind studies proved it as an effective therapeutic option	([12, 18, 23])

The influence of suggestibility on tremor amplitude, frequency or direction should be included in the clinical examination. In functional disorders cognitive behavioral therapy should be the first line treatment irrespective of the evidence of additional psychiatric symptoms [15].

Only 2 studies reviewing drug influence on functional tremor were found. These reviews discussed the usage of drugs on a low uncontrolled level. Randomized and blinded trials are not available.

A significant decrease of the Psychogenic Movement Disorder Rating Scale score could be detected after active but not sham repetitive transcranial magnetic stimulation at a rate of 1 Hz and an intensity of 90% of the resting motor threshold of the first dorsal interosseous or the tibialis anterior muscle. This effect still existed 12 months after the start of the invention. A relevant effect of hypnosis could not be shown [31] (HR).

In a single blinded study 33 patients with different functional movement disorders undergoing repeated low-frequency (0.25 Hz) magnetic stimulation over the motor cortex contralateral to the maximal symptom side or the spinal roots (RMS = root magnetic stimulation) on the symptom side showed a 66% symptom improvement both for RMS and TMS. The physicians who were rating the symptom severity were blinded to the stimulation technique. Apart from a placebo effect the authors discussed a cognitive-behavioral effect; they were skeptical concerning neuromodulation effect which is not to be expected at 0.25 Hz anyhow [13] (HR).

Symptoms of 24 patients with functional movement disorders were blindly video scored before and after a low frequency (0.25 Hz) TMS stimulation over the motor cortex contralateral to the symptoms. In 75% of the patients, the overall score improved more than 50% with a sustaining effect in a follow-up in median 19.8 months afterwards [14] (HR).

Dafotakis et al. tested 11 patients with functional tremor of the upper limb with a 0.2 Hz TMS protocol that applied 30 TMS pulses over the hand area of the primary motor cortex contralateral to the affected side documented by kinematic motion analysis. A significant reduction of tremor frequency could be documented in all patients. The authors conclude, that TMS might be an effective tool in the treatment of functional tremor, because the patients can have the expedience that the tremor also can stop, supporting the idea of a functional origin. In the context of all uncontrolled rTMS studies one might argue that the longer the aftereffects last the more a psychogenic pathogenesis might be assumed [9] (MR).

A typical clinical feature of functional tremor is the tremor entrainment in combination with or without the inability to perform simple cued movements with an unaffected body part. One study aimed to use this feature not only as a diagnostic but also as a therapeutic tool, in detail as a biofeedback treatment named ‘retrainment’. Biofeedback should teach patients the self-modulation of the tremor frequency as well as the severity of their tremor, eventually leading to volitional control of the movements of the affected body parts. Tremor entrainment was evaluated by tactile and auditory external cueing and real-time visual feedback on a computer screen. A short-term treatment was done of 2 hours, extended for up to two additional sessions until entrainment occurred. Of 10 patients with functional tremor, 3 patients had complete tremor freedom until the last follow-up visit after 6 months and a tremor improvement up to 6 months was seen in 6 patients, with 4 of them having relapses afterwards [11] (MR).

In addition to the validated and established cognitive behavioral therapy (CBT) other psychiatric concepts have been explored in clinical trials. Two thirds of patients with functional movement disorders that underwent Psychodynamic Psychotherapy (PDP) retrospectively showed some benefit. The aim of PDP is to explore potential underlying psychopathology and psychological conflicts which possibly show a connection to the neurological symptoms. This may help resolving the dysfunctional processes. Nevertheless, this treatment is time consuming and expensive and should be considered in some patients not responding to cognitive behavioral therapy which is the most standardized and evaluated kind of psychological treatment [12, 29] (HR/LR). This methods can be tentatively supplemented by relaxation techniques and physiotherapy [23] (LR), in particular in children and adolescents [18] (MR).

## Case report

The differential diagnosis is outlined in the context of a patient with a strong disabling tremor raising the suspicion of a functional origin, but also with a history of a peripheral nerve trauma of the same limb, being mentioned as a possible cause of tremor as well [6]. A 48-year-old patient complained about a progressive tremor of the right arm (first occurrence August 2016) with the strongest expression in the right hand. Four years before the start of the tremor a giant cell tumor in the distal right radius had been surgically removed (December 2012), preceded by increasing chronic pain for a few months. Pain with varying severity continued over the years in the tumor area. Drug therapy resistance of the pain led to other procedures including a pain catheter implantation inducing a transient complete anesthesia of the right arm for several months. After multiple infiltrations the tremor occurred for the first time. In the further course repeated surgical procedures (including a cancellous bone replacement/implantation of foreign material, removing of a neurinoma), were carried out and a resection of the superficial ramus of the radial nerve was required because of scarred lesions (February 2018). A carpal tunnel syndrome was excluded (unremarkable findings in neurographies, August 2018.) In the immediate temporal context of this investigation the patient claimed a deterioration of the tremor existed since then all day.

At presentation the patient complained about persistent burning pain with a pronounced painful sensitivity to touch in the hand and forearm (fulfilling allodynia criteria) why she could hardly use her hand in all day activities. Tremor and pain increased significantly with tension or physical stress. No improvement of the tremor with alcohol and no family history of tremor was reported. Typical drug therapy attempts (pregabalin, gabapentin, tilidine, capsaicin in topical use in high dosages, increased to the tolerance level until side effects occurred) could not achieve full pain relief and had no influence on the tremor. Outpatient physiotherapy was carried out with a residual pain medication (transdermal buprenorphine 10 μ / h and naproxen 500 mg on demand up to 1000 mg/d).

The patient had a coarse high-amplitude, most likely middle-rate (about 4–6 / s) permanent tremor of the distal arm in the elbow joint with involvement of the proximal arm when elevated *(Video Examination Part 1–4).* Nevertheless, typical CRPS features such as sudomotor/vasomotor dysfunctions and motor and trophic signs were not evident [17, 30]. Further, laboratory diagnostics including copper and ceruloplasmin were normal.

By performing movements with the contralateral body side, the symptoms were distractible, further, movements of the contralateral arm showed co-activation of the hand with an increase in tremor intensity *(Video Examination Part 1–4)*. Repeated painful surgery on the extremity would require the discussion of a Complex Regional Pain Syndrome (CRPS), but the complete absence of trophic disturbances is incongruous. Case reports of tremor have been described but only after markable deafferentation not fitting well in this case [24]. There were no signs of bradykinesia and Parkinson’s syndrome, nor evidence of essential or enhanced physiological tremor. For resting as well as holding and action tremor and lack of connection to the orthostasis a dystonic tremor had been discussed, but analogical symptoms of the extremities and the rest of the body could not be found. The chronic pain and the resulting pronounced psychosocial stress were also considered as a cause or at least trigger. During an additional inpatient stay, neurophysiological recordings were carried out, which showed no abnormalities. Furthermore, a precise tremor analysis with determination of the amplitude and the constancy of the frequency was performed, including the examination of an entrainment sign with a decrease in the amplitude or irregularity under distraction or repetitive voluntary movement *(Video Entrainment_Coactivation).*

The patient stayed for 24 h in the Video-EEG Monitoring unit, including recording of the electrical activity of right arm muscles using surface electrodes, at that, an irregular tremor, mostly of the right hand, partially of the entire right arm was observed. It showed a relatively high frequency, with irregularities, at times combined with myoclonic jerks. A passive fixation of the hand led to an increase of the symptoms in the arm and in the shoulder. This phenomenon is described as “whack-a-mole” sign in patients with functional movement disorders *(Video Entrainment_Coactivation)* [24].

During the examination of the right arm, the movement disorder increased with a spontaneous swinging of the right arm *(Video Examination Part 1–4).* While performing activities of the left arm such as drinking from a cup or creaming the face the tremor stopped in the meantime. The right hand was used intermittently during activities, e.g. to spread butter on bread, as well as using a cosmetic item *(Video Part 6/7 Eating).* During sleep the tremor stopped completely and resumed after waking up. Overall, due to the irregular character, clearly situationally enhanced tremor and the clear suspension in distraction, a functional genesis of the movement disorder is assumed.

The patient had a long history of multiple surgical procedures, severe pain and associated mental distress. However, psychiatric disorders such as anxiety and panic disorder or depression were not documented. CRPS is a pain disorder affecting the limbs, which can be triggered by limb trauma either without (CRPS type I), or with a definable nerve lesion (CRPS type II). Patients complain about painful sensations, allodynia, hyperalgesia and usually show autonomic dysfunction like sudomotor disturbances and temperature changes. Further, this can be combined with motor dysfunction [17]. The radial nerve lesion, the multiple interventions, motoric dysfunctions as well as the severe pain could link to a CRPS. However, the autonomic dysfunctions were missing. Basal ganglia dysfunction is reported in complex regional pain syndrome, and clinical case series reported tremor in 16 up to 49% of CRPS-I patients [3]. Quantitative investigations of postural hand tremor in CRPS patients (reported in 57% of the cases) showed similarities with physiological tremor and concluded that tremor in CRPS is an enhanced form of this [10]. On the other hand, several clinical and neuroimaging studies pointed towards dysfunction of the basal ganglia in CRPS patients [3]. A recent MRI-study showed a bilateral decrease in gray matter density in the putamen of CRPS patients. Additionally higher levels of pain and motor impairment were associated with an increased functional connectivity between the putamen and the pre−/postcentral gyri [4]. Based on these results there was evidence of a pathophysiological involvement of the basal ganglia for the development of clinical motor symptoms in CRPS. Today, after 4 years, the patient continues to experience the described symptoms of tremor, pain and allodynia. The expression fluctuates between individual days.

## Conclusion

In summary we think that the tremor in this patient is functional. Due to the distractibility of the tremor, intermittent good function and the whack a mole sign, a functional genesis is most likely. Unusual is the high level of symptoms throughout the day and in situations where the patient feels unobserved, the absence of tremor in sleep is however not differentiating. The pain and sensory disturbances make it difficult to examine and classify the symptoms. Due to this complex situation, a definitive classification of the tremor could be based on objective diagnostic criteria within the scope of full-time observation using Video-Monitoring and documentation.

## Supplementary information

**Additional file 1:** Part 1 Examination.

**Additional file 2:** Part 2 Examination.

**Additional file 3:** Part 3 Examination.

**Additional file 4:** Part 4 Examination.

**Additional file 5:** Part 5. Reading.

**Additional file 6:** Part 6. Eating.

**Additional file 7:** Part 7. Eating.

**Additional file 8:** Entrainment_Coactivation.

**Additional file 9.** Supplementary material.

## Data Availability

The datasets used and analysed during the current study are available from the corresponding author on reasonable request.

## Abbreviations

CRPS: Complex Regional Pain Syndrome
ET: Essential tremor
FMD: Functional movement disorder
FT: Functional tremor
FTT: Finger Tapping Test
PMD: Psychogenic movement disorder
rCBF: Relative cerebral blood flow
TMS: Transcranial magnetic stimulation
