# Supplementary material for: Diagnosis and therapy of functional tremor a systematic review illustrated by a case report
Source: Neurol Res Pract. 2020 Dec 3;2:35. doi: 10.1186/s42466-020-00073-1 (PMC7713151; doi:10.1186/s42466-020-00073-1)
Supplement: Supplementary file 9 — Additional file 9. Supplementary material. [file 42466_2020_73_MOESM9_ESM.docx]

Supplementary material

grey = research and diagnostic

red = therapy studies

green = Case reports

blue = overview

| Study | Authors/Journal | Year | Participants | Control Group | Healthy Controls | Abstract | Rating |
| --- | --- | --- | --- | --- | --- | --- | --- |
| Validation of „laboratory-supported“ criteria for functional (psychogenic) tremor. | 1. Mov Disord. 2016 Apr;31(4):555-62. doi: 10.1002/mds.26525. Epub 2016 Feb 16.  Validation of "laboratory-supported" criteria for functional (psychogenic)  tremor.  Schwingenschuh P(1)(2), Saifee TA(3), Katschnig-Winter P(1), Macerollo A(3),  Koegl-Wallner M(1), Culea V(1), Ghadery C(1), Hofer E(1)(4), Pendl T(1), Seiler  S(1), Werner U(1), Franthal S(1), Maurits NM(5), Tijssen MA(5), Schmidt R(1),  Rothwell JC(3), Bhatia KP(3), Edwards MJ(3)(6). | 2016 | 111 |  |  | A battery of electrophysiological tests to distinguish psychogenic from organic tremor was composed and tested. 38 patients with functional and 73 patients with organic tremor were tested and their tremor at rest and while doing different movements was judged by raters not knowing the initial diagnosis. With a sensitivity of 89.5 %, a specificity of 95.9 % and good interrater and test-retest reliability this battery can be used for diagnosing and distinguishing different forms of tremor. | HR |
| Moving toward „ laboratory-supported“ criteria for psychogenic tremor. | 1. Mov Disord. 2011 Dec;26(14):2509-15. doi: 10.1002/mds.23922. Epub 2011 Sep 28.  Moving toward "laboratory-supported" criteria for psychogenic tremor.  Schwingenschuh P(1), Katschnig P, Seiler S, Saifee TA, Aguirregomozcorta M,  Cordivari C, Schmidt R, Rothwell JC, Bhatia KP, Edwards MJ. | 2011 | 38 |  |  | Characteristics of psychogenic tremor and organic tremor and their occurrence within different electrophysiological tests were collected and differences assessed. Differentiation between both tremor forms with high certainty requires a combination of several tests including for example entrainment, frequency changing with tapping, increase in amplitude with loading or tonic coactivation at tremor onset. The authors composed a combination of tests to distinguish psychogenic from organic tremor. | HR |
| Wavelet coherence analysis: A new approach to distinguish organic and functional tremor  types. | 1: Kramer G, Van der Stouwe AMM, Maurits NM, Tijssen MAJ, Elting JWJ. Wavelet  coherence analysis:  A new approach to distinguish organic and functional tremor  types.  Clin Neurophysiol. 2018 Jan;129(1):13-20. doi:  10.1016/j.clinph.2017.10.002. Epub 2017 Oct 14. PMID: 29136548. | 2018 |  |  |  | Wavelet coherence analysis (WCA) enables detection of over time coherence variations and phase differences between signals. In this retrospective study polymyography recordings of the upper and fore arm muscles of 98 tremor patients (26 Parkinsonian (PT), 26 functional (FT), 26 essential (ET), 20 enhanced physiological (EPT) tremor) were analysed in regard of coherence and phase related parameters. Using the area under the receiver operating characteristic curve (AUC-ROC) WCA was compared with standard coherence analysis. Discriminative parameters could be used to distinguish organic from functional tremor with a higher AUC-ROC value for WCA than standard coherence analysis, concluding that the method could be an effective tool in tremor differential diagnostics. | HR |
| Usefulness of intermuscular coherence and cumulant analysis in the diagnosis of postural tremor. | 1: van der Stouwe AM, Conway BA, Elting JW, Tijssen MA, Maurits NM.  Usefulness  of intermuscular coherence and cumulant analysis in the diagnosis of postural  tremor.  Clin Neurophysiol. 2015 Aug;126(8):1564-9. doi:  10.1016/j.clinph.2014.10.157. Epub 2014 Nov 8. PMID: 25432424. | 2015 |  |  |  | Coherence and synchronicity measurements using EMG recordings from muscle pairs (wrist and elbow extensors) can help to assess postural upper-limb tremor in different patient groups. The intermuscular coherence was higher in Parkinson patients than in patients suffering from functional and essential tremor. Lowest values were obtained in enhanced physiological tremor. Further, the synchronicity pattern was higher in Parkinson tremor and functional tremor than in the essential one. | HR |
| SPECT perfusion patterns distinguish psychogenic from essential tremor. | 1. Parkinsonism Relat Disord. 2011 Jun;17(5):328-32. doi:  10.1016/j.parkreldis.2011.01.012. Epub 2011 Feb 12.  SPECT perfusion patterns distinguish psychogenic from essential tremor.  Czarnecki K(1), Jones DT, Burnett MS, Mullan B, Matsumoto JY. | 2011 | 10 | 5 |  | Cerebral perfusion patterns in essential and psychogenic tremor compared to healthy controls were examined during rest and a tremor-inducing motor task. During rest and motor task both tremor forms showed different areas with increased blood flow by which they can be distinguished. | HR |
| Sensitivity and specificity of the finger tapping task for the detection of psychogenic movement disorders. | 1. Parkinsonism Relat Disord. 2010 Mar;16(3):197-201. doi:  10.1016/j.parkreldis.2009.11.007. Epub 2009 Dec 14.  Sensitivity and specificity of the finger tapping task for the detection of  psychogenic movement disorders.  Criswell S(1), Sterling C, Swisher L, Evanoff B, Racette BA. | 2010 | 195 | 130 |  | Finger tapping test can be used supporting the detection of psychogenic movement disorders. Test scores of 195 patients with a movement disorder like idiopathic Parkinson’s disease, essential tremor or psychogenic movement disorders were evaluated and compared to those of 130 healthy controls. Scores of patients with psychogenic abnormalities were generally lower compared with other diagnostic groups. | HR |
| Impact of DAT-SPECT on Management of Patients Suspected Parkinsonism. | 1. Clin Nucl Med. 2018 Oct;43(10):710-714. doi: 10.1097/RLU.0000000000002240.  Impact of DAT-SPECT on Management of Patients Suspected of Parkinsonism.  Mirpour S, Turkbey EB, Marashdeh W, El Khouli R, Subramaniam RM. | 2018 | 173 |  |  | The authors retrospectively reviewed DAT-SPECT scans of 173 patients suspected of parkinsonism. Most scans were ordered to either distinguish parkinsonism from other diseases like Parkinson disease, essential or psychogenic tremor or to get certainty in clinical symptoms. The scan results often affected further diagnostic procedure or treatment decisions. | HR |
| Sensory Attenuation Assessed by Sensory Evoked Potentials in Functional Movement  Disorders. | 1. PLoS One. 2015 Jun 19;10(6):e0129507. doi: 10.1371/journal.pone.0129507.  eCollection 2015.  Sensory Attenuation Assessed by Sensory Evoked Potentials in Functional Movement  Disorders.  Macerollo A(1), Chen JC(2), Pareés I(2), Kassavetis P(2), Kilner JM(2), Edwards  MJ(2). | 2015 | 17 |  | 17 | SEP amplitudes of 17 patients with a functional movement disorder at the onset of self-paced movement were measured and compared to healthy controls. Reduction of amplitude was seen in healthy controls whereas reduced sensory attenuation was observed in the patients. Conclusion was that patients with FMD have an impairment of sense of agency for movement. | HR |
| Psychogenic parkinsonism: clinical spectrum and diagnosis. | 1. Ann Clin Psychiatry. 2015 Feb;27(1):33-8.  Psychogenic parkinsonism: clinical spectrum and diagnosis.  Sage JI(1), Mark MH. | 2015 | 36 |  |  | Clinical symptoms, concomitant diseases and treatment perspectives of psychogenic parkinsonism were identified and are describes in this study. Therefore clinical data were retrospectively reviewed finding 36 patients with the diagnosis of psychogenic parkinsonism. | MR |
| Dopamine transporter imaging in psychogenic parkinsonism and neurodegenerative parkinsonism with psychogenic overlay: a report of three cases. | 1. Tremor Other Hyperkinet Mov (N Y). 2013 Sep 10;3. pii: tre-03-188-4324-2. doi:  10.7916/D8FB51PS. eCollection 2013.  Dopamine transporter imaging in psychogenic parkinsonism and neurodegenerative  parkinsonism with psychogenic overlay: a report of three cases.  Umeh CC(1), Szabo Z, Pontone GM, Mari Z. | 2013 | 3 |  |  | Psychogenic parkinsonism and neurodegenerative Parkinson’s disease can easily be confounded. Diagnostics and differentiation of these two abnormalities can be supported by using DAT-SPECT leading to certain results. | MR |
| Attention in Parkinson’s disease mimicking suggestion in psychogenic movement disorder. | 1. J Mov Disord. 2012 Oct;5(2):53-4. doi: 10.14802/jmd.12012. Epub 2012 Oct 30.  Attention in Parkinson's disease mimicking suggestion in psychogenic movement  disorder.  Baik JS(1). | 2012 | 1 |  |  | In this case one patient with parkinsonian features suspected as a psychogenic movement disorder is described. For this several characteristics that help to distinguish organic from psychogenic origins are defined. | MR |
| Electrophysiological evaluation of psychogenic movement disorders. | 1. Parkinsonism Relat Disord. 2016 Jan;22 Suppl 1:S153-8. doi:  10.1016/j.parkreldis.2015.09.016. Epub 2015 Sep 9.  Electrophysiological evaluation of psychogenic movement disorders.  Kamble NL(1), Pal PK(2). | 2016 |  |  |  | For diagnosing a psychogenic movement disorder several electrophysiological tests can be performed including accelerometry, surface electromyography, EEG, TMS or evoked potentials. These tests and their results for different psychogenic movement disorders are described. Electrophysiological methods are most useful for psychogenic tremor and less for patients with mixed pathophysiology. | HR |
| Clinical neurophysiology of psychogenic movement disorders: how to diagnose psychogenic tremor and myoclonus. | 1. Neurophysiol Clin. 2014 Oct;44(4):417-24. doi: 10.1016/j.neucli.2013.08.014. Epub  2013 Sep 13.  Clinical neurophysiology of psychogenic movement disorders: how to diagnose  psychogenic tremor and myoclonus.  E.Apartis | 2014 |  |  |  | Electrophysiological test and criteria to diagnose psychogenic movement disorders, here especially psychogenic tremor and myoclonus are provided. These test and their results should affect and define further therapeutic approaches. Clinical features of tremor are more in different cases, but the clinical presentation of myoclonus is very heterogeneous. | MR |
| Psychogenic movement disorders. | 1. Parkinsonism Relat Disord. 2012 Jan;18 Suppl 1:S155-7. doi:  10.1016/S1353-8020(11)70048-7.  Psychogenic movement disorders.  Hallett M(1), Weiner WJ, Kompoliti K. | 2012 |  |  |  | Paroxysmal nature, variability of tremor direction, distractibility and further clinical characteristics of psychogenic movement disorders are specified in this study. These features are characterized for different disorders, psychogenic parkinsonism as a difficult diagnosis is further specified. | MR |
| A practical guide to the differential diagnosis of tremor. | 1. Postgrad Med J. 2011 Sep;87(1031):623-9. doi: 10.1136/pgmj.2009.089623. Epub 2011  Jun 20.  A practical guide to the differential diagnosis of tremor.  Alty JE(1), Kempster PA. | 2011 |  |  |  | The study describes the clinical manifestations of different forms of tremor, such as frequency and amplitude of the tremor and its significance in Parkinson’s disease. It also highlights the importance of ruling out other and more benign conditions that present with resting tremor. | LR |
| Electrophysiologic evaluation of psychogenic movement disorders. | 1. J Mov Disord. 2011 May;4(1):21-32. doi: 10.14802/jmd.11004. Epub 2011 Apr 30.  Electrophysiologic evaluation of psychogenic movement disorders.  Pal PK(1). | 2011 |  |  |  | This study describes different methods, like EMG or accelerometry, to diagnose different forms of psychogenic movement disorders (PMD). Several laboratory investigations and electrophysiological tests should be done to rule out organic disorders and to differentiate between the different movement abnormalities. Those are the psychogenic tremor, psychogenic jerks, spasms, and dystonia. | HR |
| Functional tremor. | 1. Handb Clin Neurol. 2016;139:229-233. doi: 10.1016/B978-0-12-801772-2.00019-9.  Functional tremor.  Schwingenschuh P(1), Deuschl G(2). | 2016 |  |  |  | Diagnosing functional tremor with high certainty several criteria like sudden onset, fluctuations or distractibility need to be considered. In difficult cases and also to distinguish psychogenic tremor from Parkinson’s disease laboratory-supported criteria such as DAT-SPECT can be helpful. | MR |
| Diagnosis and Treatment of Functional (Psychogenic) Parkinsonism. | 1. Semin Neurol. 2017 Apr;37(2):228-232. doi: 10.1055/s-0037-1601487. Epub 2017 May  16.  Diagnosis and Treatment of Functional (Psychogenic) Parkinsonism.  LaFaver K(1), Espay AJ(2). | 2017 |  |  |  | Clinical features of functional parkinsonism are described as well as other functional phenotypes like functional tremor, which may coexist. DAT scan can be used in addition to standardized test to diagnose psychogenic parkinsonism with high certainty. Also treatment approaches are mentioned. | MR |
| Psychogenic (functional) parkinsinism. | 1. Handb Clin Neurol. 2016;139:259-262. doi: 10.1016/B978-0-12-801772-2.00022-9.  Psychogenic (functional) parkinsonism.  Thenganatt MA(1), Jankovic J(2). | 2016 |  |  |  | Psychogenic parkinsonism is the content of this study. Clinical features and how this disease can be distinguished from idiopathic Parkinson’s disease including the use of functional imaging are described. | MR |
| Repetitive transcranial magnetic stimulation for functional tremor: A randomized, double-blind, controlled study. | Taib S, Ory-Magne F, Brefel-Courbon C, Moreau Y, Thalamas C, Arbus C, Simonetta-Moreau M.  Mov Disord. 2019 Aug;34(8):1210-1219. doi: 10.1002/mds.27727. Epub 2019 Jun 10 | 2019 | 18 (33 screened) |  |  | The effect of repetitive transcranial magnetic stimulation on functional tremor was investigated in this study. 18 patients (8 men, 10 women) out of 33 screened patients fulfilled the inclusion criteria and were randomized. One group received active, the control-group sham repetitive transcranial magnetic stimulation on five consecutive daily sessions. During the second phase all participants underwent three weekly sessions of hypnosis in addition to single sessions of real repetitive transcranial magnetic stimulation. The decrease of the Psychogenic Movement Disorder Rating Scale score was significant (p < 0.001) in the group receiving the active repetitive transcranial magnetic stimulation one and two months after the intervention. The decrease of this score as well as Tremor subscores remained significant at month 6 and 12. For the control-group the Psychogenic Movement Disorder Rating Scale score decreased after one month and returned to baseline after month 2. | HR |
| Clinical and neural responses to cognitive behavioral therapy for functional tremor. | Neurology. 2019 Nov 5;93(19):e1787-e1798. doi: 10.1212/WNL.0000000000008442.  Epub 2019 Oct 4.  Espay AJ(1), Ries S(2), Maloney T(2), Vannest J(2), Neefus E(2), Dwivedi AK(2),  Allendorfer JB(2), Wulsin LR(2), LaFrance WC(2), Lang AE(2), Szaflarski JP(2). | 2019 | 15 | 15 | 25 | The effect of Cognitive Behavioral Therapy on severity and motor/emotion-processing circuits in patients with functional tremor was investigated in this study. 15 Patients with tremor underwent fMRI with motor, basic-emotion, and intense-emotion tasks before and after 12 weeks of CBT. The results were compared to 25 healthy controls. The results show significant improrvement in tremor severity in 73.3 % of the patients, which was associated with changes in the anterior cingulate/paracingulate activity. | HR |
| Impact of Transcranial Magnetic Stimulation on Functional Movement Disorders: Cortical Modulation or Behavioral Effect? | Garcin B, Mesrati F, Hubsch C, Mauras T, Iliescu I, Naccache L, Vidailhet M, Roze E, Degos B.  Front Neurol. 2017 Jul 19;8:338. doi: 10.3389/fneur.2017.00338. eCollection 2017. | 2017 | 33 |  |  | 33 patients with functional movement disorders were studied to determine if the improvement achieved with transcranial magnetic stimulation is due to neuromodulation or cognitive-behavioral effects. Half of the patients received root magnetic stimulation homolateral to the symptoms on the first day and TMS on the second day. The other half was treated vice versa. Before and after the stimulation the severity of the movement disorders was assessed blindly. Independent of the order of the treatment 22 patients received significant improvement. | HR |
| Psychodynamic Psychotherapy for Functional (Psychogenic) Movement Disorders. | Sharma VD, Jones R, Factor SA.  J Mov Disord. 2017 Jan;10(1):40-44. doi: 10.14802/jmd.16038. Epub 2016 Dec 27. | 2017 | 30 |  |  | Psychodynamic Psychotherapy (PDP) as treatment for functional movement disorders was assessed retrospectively analyzing the data of 30 patients, who underwent PDP between 2008 and 2014 at Emory University Medical Center. The mean age of the patients was 50 years, most of them were female. The average number of PDP visits was 4.9. Improvements were seen in 60 % of the patients and two patients lost to follow up. Psychological approaches are important and can be used as treatment for heterogeneous causes of functional movement disorders. To predict who will benefit from PDP and achieve improvements remains a challenge. | HR |
| Tremor retrainment as therapeutic strategy in psychogenic (functional) tremor. | 1. Parkinsonism Relat Disord. 2014 Jun;20(6):647-50. doi:  10.1016/j.parkreldis.2014.02.029. Epub 2014 Mar 20.  Tremor retrainment as therapeutic strategy in psychogenic (functional) tremor.  Espay AJ(1), Edwards MJ(2), Oggioni GD(3), Phielipp N(4), Cox B(5),  Gonzalez-Usigli H(5), Pecina C(5), Heldman DA(6), Mishra J(5), Lang AE(4). | 2014 | 10 |  |  | Ten patients with psychogenic tremor participated in the study to evaluate the feasibility of tremor entrainment as a therapeutic strategy. Tactile and auditory external cueing and real-time visual feedback on a computer screen facilitated the retraining of tremor frequency. As measured by the Tremor subscale of the Psychogenic Movement Disorder Rating Scale the tremor improved between one week and six months in six patients with four patients having relapses between week two and six months. Tremor freedom was achieved by three patients. | MR |
| Transcranial magnetic stimulation as an efficient treatment for psychogenic movement disorders. | 1. J Neurol Neurosurg Psychiatry. 2013 Sep;84(9):1043-6. doi:  10.1136/jnnp-2012-304062. Epub 2013 Feb 5.  Transcranial magnetic stimulation as an efficient treatment for psychogenic  movement disorders.  Garcin B(1), Roze E, Mesrati F, Cognat E, Fournier E, Vidailhet M, Degos B. | 2013 | 24 |  |  | Therapeutic effect of repeated transcranial magnetic stimulation on psychogenic movement disorders was investigated in this study. The symptoms of 24 patients were scored blindly before and after treatment with TMS at low frequency (0.25 Hz). The score improved by over 50 % in 75 % of the patients and benefits remained for a median of 19.8 months. | HR |
| Transcranial magnetic stimulation for psychogenic tremor - a pilot study. | Dafotakis M, Ameli M, Vitinius F, Weber R, Albus C, Fink GR, Nowak DA.  Fortschr Neurol Psychiatr. 2011 Apr;79(4):226-33. doi: 10.1055/s-0029-1246094. Epub 2011 Apr 8. German. | 2011 | 11 |  |  | Transcranial magnetic stimulation as a tool to establish the diagnosis of psychogenic hand tremor with a high certainty, to reduce tremor intensity and to facilitate the patients insight into the psychogenic origin of the movement disorder was tested in 11 patients. Seven patients achieved a transient, four patients achieved lasting symptom relief after the TMS procedure. The effectiveness was measured by kinematic motion analysis. | MR |
| The treatment of tremor. | 1. Neurotherapeutics. 2014 Jan;11(1):128-38. doi: 10.1007/s13311-013-0230-5.  The treatment of tremor.  Schneider SA(1), Deuschl G. | 2014 |  |  |  | Literature on treating different forms of tremor like essential, psychogenic, orthostatic or dystonic tremor was reviewed. The authors summarize the most helpful treatment for each tremor, which are antidepressants for psychogenic tremor for example, but emphasize the need for further studies on this topic. | LR |
| Holmes' or functional tremor? | Bocci T, Ardolino G, Parenti L, Barloscio D, De Rosa A, Priori A, Sartucci F.  Clin Neurophysiol Pract. 2018 Apr 21;3:104-106. doi: 10.1016/j.cnp.2018.03.006. eCollection 2018. | 2018 | 1 |  |  | A case of a man with combined resting-postural-kinetic tremor commonly defined as an organic disease is reported. After extensive electrophysiological assessment and neuroimaging revealing an intracranial dermoid cyst at the right pontocerebellar angle with brainstem dislocation a psychogenic genesis of the tremor is suggested. The authors prompt to re-define diagnostic criteria in hyperkinetic movement disorders to renew the distinction between organic and psychogenic movement disorders. | LR |
| Possible Functional Movement Toes Syndrome. | Vanegas-Arroyave N, Panyakaew P, Lamichhane D, Shulman L, Hallett M.  Tremor Other Hyperkinet Mov (N Y). 2016 Mar 22;6:352. doi: 10.7916/D8CZ36XT. eCollection 2016. | 2016 | 2 |  |  | The classical description of the moving toes syndrome as an organic movement disorder is scrutinized and complemented by the functional etiology of the disorder. Two patients with moving toes syndrome are described with clinical symptoms such as entrainability and distractibility typically indicating a functional movement disorder. | MR |
| Post-traumatic shoulder movement disorders: A challenging differential diagnosis between organic and functional. | 1. Mov Disord Clin Pract. 2014 Jun 1;1(2):102-105.  Post-traumatic shoulder movement disorders: A challenging differential diagnosis  between organic and functional.  Pandey S(1), Nahab F(2), Aldred J(3), Nutt J(4), Hallett M(5). | 2014 | 3 |  |  | The case report is about three patients who developed shoulder movement disorders after trauma to the shoulder region. The differentiation between functional etiology and organic was inconclusive. | LR |
| Fixed dystonia of the left hand in a volinist: a rare functional disorder. | 1. Tremor Other Hyperkinet Mov (N Y). 2013 Aug 26;3. pii: tre-03-184-4272-1. doi:  10.7916/D8K35SCP. eCollection 2013.  Fixed dystonia of the left hand in a violinist: a rare functional disorder.  Lee A(1), Jahnke AK, Altenmüller E. | 2013 | 1 |  |  | Fixed dystonia in a 21-year-old violinist after a minor injury is described. This case study states the importance of a biopsychosocial therapeutic approach, for the reason that the psychogenic disorder disappeared after injection with botulinumtoxin. | LR |
| Usefulness of the coherence entertainment test for deep brain stimulation for a patient with atypical tremor. | 1. J Clin Neurosci. 2013 Aug;20(8):1161-2. doi: 10.1016/j.jocn.2012.09.029. Epub  2013 May 7.  Usefulness of the coherence entrainment test for deep brain stimulation for a  patient with atypical tremor.  Hasegawa Y(1), Yamada K, Uekawa K, Hamasaki T, Fujise N, Kuratsu J. | 2013 | 1 |  |  | After surgery a man had onset of a resting and action right-hand tremor. A coherence entrainment test was performed revealing a non-psychogenic origin, so that further treatment strategies could be determined. | LR |
| Metabolic hyperactivity of the medial posterior parietal lobes in psychogenic tremor. | 1. Tremor Other Hyperkinet Mov (N Y). 2012;2. pii: tre-02-50-441-1. doi:  10.7916/D87W69X8. Epub 2012 May 11.  Metabolic hyperactivity of the medial posterior parietal lobes in psychogenic  tremor.  Hedera P(1). | 2012 | 1 |  |  | A positron emission tomography (PET) of brain was performed on a patient with psychogenic tremor. The 18F-deoxyglucose uptake increased symmetrically in both posterior medial parietal lobes, whereas no corresponding structural abnormality could be identified. The bilateral hypermetabolism may suggest abnormal sensory integration and a correlation between functional brain abnormalities and psychogenic movement disorders. | LR |
| A case of intractable psychogenic essential palatal tremor. | 1. J Mov Disord. 2012 Oct;5(2):55-6. doi: 10.14802/jmd.12013. Epub 2012 Oct 30.  A case of intractable psychogenic essential palatal tremor.  Chung EJ(1), Jung H(2), Kim SJ(1). | 2012 | 1 |  |  | The case report describes a patient with intractable psychogenic essential palatal tremor, a rare movement disorder. Here muscles of the soft palate show rhythmic involuntary movements. | LR |
| Astasia-abasia and psychogenic tremor post-temporal lobectomy. | 1. Epilepsy Behav. 2012 Apr;23(4):503-4. doi: 10.1016/j.yebeh.2011.12.013. Epub 2012  Mar 3.  Astasia-abasia and psychogenic tremor post-temporal lobectomy.  Arabi MR(1), Wazne J, Nasreddine W, Najjar M, Beydoun A. | 2012 | 1 |  |  |  | LR |
| Severe psychogenic tremor of both wrists in a 13-year-old girl treated successfully with a customized wrist brace: a case report. | 1. J Med Case Rep. 2011 Apr 20;5:158. doi: 10.1186/1752-1947-5-158.  Severe psychogenic tremor of both wrists in a 13-year-old girl treated  successfully with a customized wrist brace: a case report.  Sauerhoefer E(1), Schafflhuber C, Kratz O. | 2011 | 1 |  |  | After complete neurological and psychiatric examination cognitive-behavioral therapy was performed with a 13-year old girl with psychogenic tremor in both wrists. Chronic and acute stress factors were addressed during therapy and led to a prompt reduction of 80 % in tremor severity. Two weeks after psychotherapy the girl achieved complete remission. | LR |
| Gender Differences in Functional Movement Disorders. | Mov Disord Clin Pract. 2019 Dec 24;7(2):182-187. doi: 10.1002/mdc3.12864.  eCollection 2020 Feb  Gender Differences in Functional Movement Disorders.  Baizabal-Carvallo JF(1)(2), Jankovic J(1). | 2020 | 196 |  |  | This study compared clinical and functional features of functional movement disorders in 196 patients. The conclusion is that women are over-represented, especially in people under 50 years. Also functional dystonia is more common in females. | HR |
| Clinical Characteristics of Functional Movement Disorders: A Clinic-based Study. | 1. J Neurol Sci. 2010 Jan 15;288(1-2):68-71. doi: 10.1016/j.jns.2009.10.004. Epub  2009 Nov 6.  Post-streptococcal 'complex' movement disorders: unusual concurrence of  psychogenic and organic symptoms.  Squintani G(1), Tinazzi M, Gambarin M, Bravi E, Moretto G, Buttiglione M, Defazio  G, Martino D. | 2018 | 321 |  |  | 321 patients with movement abnormalities were screened to evaluate the incidence of functional movement disorders and also describe clinical and phenomenological characteristics. Tremor, especially occurring in upper and lower extremities, and speech abnormalities were most common among 31 patients diagnosed with a functional movement disorder. 61 % of them were affected by depression, anxiety or other psychiatric illnesses in the past. Patients with a mean symptom duration of 6.2 years had poorer clinical outcomes as patients with shorter duration. | HR |
| Impared emotion processing in functional (psychogenic) tremor: A functional magnetic resonance imaging study. | 1. Neuroimage Clin. 2017 Oct 18;17:179-187. doi: 10.1016/j.nicl.2017.10.020.  eCollection 2018.  Impaired emotion processing in functional (psychogenic) tremor: A functional  magnetic resonance imaging study.  Espay AJ(1), Maloney T(2), Vannest J(2), Norris MM(3), Eliassen JC(3), Neefus  E(1), Allendorfer JB(4), Lang AE(5), Szaflarski JP(4). | 2017 | 43 |  | 25 | Differences in motor and emotion networks in patients with functional tremor (27), essential tremor (16) and healthy controls (25) were examined using functional magnetic resonance imaging. Outcomes of a finger-tapping motor task, a basic-emotion task and an intense-emotion task were evaluated and compared. Patients with functional tremor showed alterations in activation and connectivity in brain areas associated with emotion processing and theory of mind compared to patients with essential tremor or healthy controls. | HR |
| Psychogenic Movement Disorders in Adults and Children: A Clinical and Video Profile of 58 Indian Patients. | 1. Mov Disord Clin Pract. 2017 Jul 18;4(5):763-767. doi: 10.1002/mdc3.12516.  eCollection 2017 Sep-Oct.  Psychogenic Movement Disorders in Adults and Children: A Clinical and Video  Profile of 58 Indian Patients.  Pandey S(1), Koul A(1). | 2017 | 58 |  |  | This report gives an overview of 58 patients with psychogenic movement disorder. 33 adults and 25 children were examined in reference to disease onset, duration, precipitating factors diagnosis and outcome as well as movement phenotype, variability, distractibility and entrainment. Most common movement disorder in adults and children was tremor and symptoms appeared abruptly in most of the patients. Precipitating factors were related to family and social issues for adults and associated with examination and school-related issues for children. | HR |
| Functional (psychogenic) stereotypies. | Baizabal-Carvallo JF, Jankovic J.  J Neurol. 2017 Jul;264(7):1482-1487. doi: 10.1007/s00415-017-8551-7. Epub 2017 Jun 26. | 2017 | 19 (184) |  |  | Different functional movement disorders including orolingual dyskinesia, limb and trunk stereotypies and respiratory dyskinesia are described and their characteristics compared with tardive dyskinesia. The 19 patients with functional stereotypies differed from the 65 patients with tardive dyskinesia in sudden onset of their symptoms , distractibility and periods of unexplained improvement. Further differences are the older age at onset and self-biting in patients with tardive dyskinesia. These characteristics help to differentiate different types of movement disorders. | HR |
| Distinguishing features of psychogenic (functional) versus organic hemifacial spasm. | 1. J Neurol. 2017 Feb;264(2):359-363. doi: 10.1007/s00415-016-8356-0. Epub 2016 Dec  9.  Distinguishing features of psychogenic (functional) versus organic hemifacial  spasm.  Baizabal-Carvallo JF(1)(2), Jankovic J(3). | 2017 | 18 |  |  | 18 patients with psychogenic and 37 with organic hemifacial spasm were examined finding features that help to distinguish both forms from one another. On average patients with a psychogenic origin were younger, showed more frequently tonic muscle contractions, a lack of the ‚other Babinski sign‘ and a downward deviation of the mouth’s angle. Besides these characteristics suggestibility, distractibility and periods of unexplained improvement can be used to differentiate psychogenic from organic. | MR |
| Gender and Age Determinants of Psychogenic Movement Disorders: A Clinical Profile of 73 Patients. | 1. Can J Neurol Sci. 2016 Mar;43(2):268-77. doi: 10.1017/cjn.2015.365. Epub 2016 Jan  13.  Gender and Age Determinants of Psychogenic Movement Disorders: A Clinical Profile  of 73 Patients.  Kamble N(1), Prashantha DK(1), Jha M(1), Netravathi M(1), Reddy YC(2), Pal PK(1). | 2016 | 73 |  |  | Neurological and psychiatric characteristics of psychogenic movement disorders in a cohort of 73 patients were examined in a period of 14 years. The most often disorder was tremor in adults and myoclonus in children, both groups with depression as the common comorbidity . 61.6 % had abrupt onset of symptoms with the upper limb as the initial affected body part. After counseling, antidepressants and placebo 57.5 % showed reduction or complete remission. | MR |
| Increased variability in spiral drawing in patients with functional (psychogenic) tremor. | 1. Hum Mov Sci. 2014 Dec;38:15-22. doi: 10.1016/j.humov.2014.08.007. Epub 2014 Sep  18.  Increased variability in spiral drawing in patients with functional (psychogenic)  tremor.  Hess CW(1), Hsu AW(1), Yu Q(1), Ortega R(2), Pullman SL(3). | 2014 | 43 |  | 31 | 22 patients with functional tremor, 21 with dystonic tremor and 31 healthy controls were compared in a spiral drawing performance in terms of spiral severity and inter-spiral variability. Patients with functional and dystonic tremor had higher severity than healthy controls. High inter-spiral variability was seen in the functional tremor group. | MR |
| Psychogenic Movement DIsorders: Gait Is a Give-Away! | 1. Mov Disord Clin Pract. 2014 May 26;1(2):110-111. doi: 10.1002/mdc3.12031.  eCollection 2014 Jun.  Psychogenic Movement Disorders: Gait Is a Give-Away!  Balint B(1)(2), van Winsen LML(3), Bhatia KP(2), Bloem BR(3). | 2014 | ? |  |  | To distinguish weather a movement disorder has a psychogenic or an organic origin, walking patterns of patients can be examined. Incongruity of the gait pattern with the particular disorder was observed in a case series of patients with different types of psychogenic movement disorders. | MR |
| A clinical study of non-parkinsonian tremor in Moroccan patients. | 1. Rev Neurol (Paris). 2014 Jan;170(1):26-31. doi: 10.1016/j.neurol.2013.06.006.  Epub 2013 Dec 7.  A clinical study of non-parkinsonian tremor in Moroccan patients.  Regragui W(1), Lachhab L(2), Razine R(3), Ait Benhaddou EH(4), Benomar A(5),  Yahyaoui M(4). | 2014 | 62 (148) |  |  | Clinical features and treatment of non-parkinsonian tremor were retrospectively analysed in 62 patients. Essential tremor was the most common form, followed by dystonic tremor. More female than male patients were affected and mean age at the onset was 52.2 years. In 94.1 % the arms were affected by tremor, symptoms were bilateral in 87.5 % but asymmetrical in half of the patients. Main treatment was based on drugs especially propanolol. | MR |
| Shell shock: Psychogenic gait and other movement disorders-A film review. | 1. Tremor Other Hyperkinet Mov (N Y). 2013;3. pii: tre-03-110-774-2. doi:  10.7916/D89023HJ. Epub 2013 Mar 28.  Shell shock: Psychogenic gait and other movement disorders-A film review.  Moscovich M(1), Estupinan D, Qureshi M, Okun MS. | 2013 | 21 |  |  | This paper reanalyzes the film footage of ‚War Neuroses‘ by Arthur Hurst. Resulting from World War I psychogenic gait and other movement disorders pre and post treatment in 21 patients shown in the film were re-examined. The impressive results of treating psychogenic disorders presented in the film were conspicuous and scrutinize its authenticity. | LR |
| Functional movement disorders are not uncommon in the elderly. | 1. Mov Disord. 2013 Apr;28(4):540-3. doi: 10.1002/mds.25350. Epub 2013 Feb 15.  Functional movement disorders are not uncommon in the elderly.  Batla A(1), Stamelou M, Edwards MJ, Pareés I, Saifee TA, Fox Z, Bhatia KP. | 2013 | 151 |  |  | The aim of the study was to report clinical characteristics of functional movement disorders in elderly people compared to patients with a younger age of onset. 33 out of 151 patients attributed to the elderly group with onset after 60 years contrary to the younger group, where onset was around 35.5 years. In both groups tremor was the most common disorder, whereas gait abnormalities and psychogenic nonepileptic seizures were more often in the elderly group. | HR |
| Psychogenic balance disorders: is it a new entity of psychogenic movement disorders? | 1. J Mov Disord. 2012 May;5(1):24-7. doi: 10.14802/jmd.12007. Epub 2012 May 30.  Psychogenic balance disorders: is it a new entity of psychogenic movement  disorders?  Baik JS(1), Lee MS(2). | 2012 | 3 |  |  | Clinical characteristics of three patients with balance or posture disorders are described. These patients differences to reported cases of astasia without abasia and of psychogenic gait disorders are discussed. | LR |
| Diagnostic challenges revealed from a neuropsychiatry movement disorder clinic. | 1: Rigby H, Roberts-South A, Kumar H, Cortese L, Jog M. Diagnostic challenges  revealed from a neuropsychiatry movement disorders clinic. Can J Neurol Sci. 2012  Nov;39(6):782-8. PubMed PMID: 23041398. | 2012 | 106 |  |  | This retrospective study reveals different assessments of phenomenology and diagnoses in patients with movement disorders and co-existing psychiatric symptoms made by a movement disorder specialist and physicians. 106 patients were reviewed to evaluate inter-rater reliability, which was highest for tardive dyskinesia and drug induced tremor but overall turned out to be poor. | MR |
| Psychogenic palatal tremor may be underrecognized: reappraisal of a larfe series of cases. | 1. Mov Disord. 2012 Aug;27(9):1164-1168. doi: 10.1002/mds.24948. Epub 2012 Mar 20.  Psychogenic palatal tremor may be underrecognized: reappraisal of a large series  of cases.  Stamelou M(#)(1), Saifee TA(#)(1), Edwards MJ(1), Bhatia KP(1). | 2012 | 17 |  |  | The occurrence of palatal tremor, especially the psychogenic form, was retrospectively investigated in a clinic over a period of 10 years. 10 patients with isolated palatal tremor were identified of which 70 % received the diagnosis of a psychogenic disorder. | LR |
| Phenomenology of psychogenic movement disorders in children. | 1. Mov Disord. 2012 Aug;27(9):1153-7. doi: 10.1002/mds.24947. Epub 2012 Mar 7.  Phenomenology of psychogenic movement disorders in children.  Canavese C(1), Ciano C, Zibordi F, Zorzi G, Cavallera V, Nardocci N. | 2012 | 14 |  |  | The study includes 14 patients unter 18 years with psychogenic movement disorders to describe clinical features of these disorders. Tremor followed by dystonia were the most common diagnoses. 71 % suffered from a single 29 % from two or more disorders. Prevalent was the presence of other psychogenic symptoms associated with the movement abnormality. | MR |
| Believing is perceiving: mismatch between self-report and actigraphy in psychogenic tremor. | 1. Brain. 2012 Jan;135(Pt 1):117-23. doi: 10.1093/brain/awr292. Epub 2011 Nov 10.  Believing is perceiving: mismatch between self-report and actigraphy in  psychogenic tremor.  Pareés I(1), Saifee TA, Kassavetis P, Kojovic M, Rubio-Agusti I, Rothwell JC,  Bhatia KP, Edwards MJ. | 2012 | 18 |  |  | The evaulations of tremor duration and severity of ten patients with psychogenic and eight with organic tremor were compared to data measured by actigraphy over a period of five days. Patients with psychogenic tremor reported significantly more tremor than recorded by actigraphy. The difference was lesser in patients with organic symptoms. Further discussions are needed to understand the discrepancy between perceived and real tremor occurrence. | MR |
| Movement disorder emergencies in childhood. | 1. Eur J Paediatr Neurol. 2011 Sep;15(5):390-404. doi: 10.1016/j.ejpn.2011.04.005.  Epub 2011 Aug 10.  Movement disorder emergencies in childhood.  Kirkham FJ(1), Haywood P, Kashyape P, Borbone J, Lording A, Pryde K, Cox M,  Keslake J, Smith M, Cuthbertson L, Murugan V, Mackie S, Thomas NH, Whitney A,  Forrest KM, Parker A, Forsyth R, Kipps CM. | 2011 | 52 |  |  | The report describes acute-onset movement disorders in children. 52 children under 15 years were analyzed. Most common disorders were chorea, dystonia and tremor. These were furthermore subclassified in psychogenic, inflammatory or autoimmune and non-inflammatory disorders. Causes of these movement abnormalities were for example trauma and severe cerebral palsy. Treatment of these movement disorders is different and relies on the underlaying cause. | LR |
| Abnormal sense of intention preceding voluntary movement in patients with psychogenic tremor. | 1. Neuropsychologia. 2011 Jul;49(9):2791-3. doi:  10.1016/j.neuropsychologia.2011.05.021. Epub 2011 Jun 6.  Abnormal sense of intention preceding voluntary movement in patients with  psychogenic tremor.  Edwards MJ(1), Moretto G, Schwingenschuh P, Katschnig P, Bhatia KP, Haggard P. | 2011 | 9 |  |  | In this study nine patients with psychogenic tremor were asked to judge their internal feeling of intention to press a self-paced button relative to a clock. The sense of volition occurred much later compared to healthy controls. Impairment of neural mechanisms responsible for conscious actions might explain these perceptions. | LR |
| Peripheral trauma and movement disorders: a systematic review of reported cases. | 1. J Neurol Neurosurg Psychiatry. 2011 Aug;82(8):892-8. doi:  10.1136/jnnp.2010.232504. Epub 2011 Apr 14.  Peripheral trauma and movement disorders: a systematic review of reported cases.  van Rooijen DE(1), Geraedts EJ, Marinus J, Jankovic J, van Hilten JJ. | 2011 | 713 |  |  | 133 publications on 713 patients were interpreted referring movement disorders provoked by peripheral trauma. Most common abnormality was fixed dystonia associated with pain and sensory disorder. Other diagnoses were regional pain syndrome and psychogenic movement disorders. The authors conclude that many movement disorders are caused by peripheral trauma and discuss the underlying mechanisms. | MR |
| Tremor in hemifacial spasm patients. | 1. J Neural Transm (Vienna). 2011 Feb;118(2):241-7. doi: 10.1007/s00702-010-0533-1.  Epub 2010 Dec 16.  Tremor in hemifacial spasm patients.  Rudzińska M(1), Wójcik M, Hartel M, Szczudlik A. | 2011 | 47 |  | 48 | To identify tremor characteristics in patients with hemifacial spasm, 47 of them were examined and compared to 48 healthy controls matching in sex and age. Tremor was identified in 19 patients and in five healthy controls. Tremor types and correlation to the severity of hemifacial spasm are specified. | MR |
| Cross-cultural influences on psychogenic movement disorders - a comparative review with a Brazilian series of 83 cases. | 1. Clin Neurol Neurosurg. 2011 Feb;113(2):115-8. doi:  10.1016/j.clineuro.2010.10.004. Epub 2010 Nov 30.  Cross-cultural influences on psychogenic movement disorders - a comparative  review with a Brazilian series of 83 cases.  Munhoz RP(1), Zavala JA, Becker N, Teive HA. | 2011 | 83 |  |  | 83 patients from Brazil with a psychogenic movement disorder were included to characterize symptoms and diagnoses and compare them with worldwide raised data. Particularly affected were females. The most common diagnosis was psychogenic tremor, followed by dystonia and 80 % of the patients have psychiatric co-morbidity. These finding coincide with former findings from international data. | MR |
| Unilateral lower limb rest tremor is not necessarily a presenting symptom of Parkinson’s disease. | 1. Mov Disord. 2010 May 15;25(7):924-7. doi: 10.1002/mds.23030.  Unilateral lower limb rest tremor is not necessarily a presenting symptom of  Parkinson's disease.  Hellmann MA(1), Melamed E, Steinmetz AP, Djaldetti R. | 2010 | 16 |  |  | To identify the variety of underlying causes of lower leg rest tremor, 16 patients were reviewed. Besides Parkinson’s disease and multiple system atrophy psychogenic tremor and drug-induced parkinsonism were also diagnosed. | MR |
| A prospective study of acuate movement disorders in children. | 1. Dev Med Child Neurol. 2010 Aug;52(8):739-48. doi:  10.1111/j.1469-8749.2009.03598.x. Epub 2010 Feb 12.  A prospective study of acute movement disorders in children.  Dale RC(1), Singh H, Troedson C, Pillai S, Gaikiwari S, Kozlowska K. | 2010 | 52 |  |  | Movement disorders in 52 children were classified in inflammatory or autoimmune disease, in non-inflammatory and in psychogenic disorders. These were further subclassified and described. Treatment showed variable results and must be adapted to each underlying cause of the movement disorder. | MR |
| Treatment of functional movement disorder. | 1. Neurol Clin. 2020 May;38(2):469-480. doi: 10.1016/j.ncl.2020.01.011. Epub 2020  Mar 9.  Treatment of Functional Movement Disorders.  LaFaver K(1). | 2020 |  |  |  | This study addresses functional movement disorders as a common diagnosis in neurologic practice and describes common symptoms seen in patients. Different treatment options are mentioned, especially a multidisciplinary therapy could benefit many patients. | LR |
| Approach to a tremor patient. | 1. Ann Indian Acad Neurol. 2016 Oct-Dec;19(4):433-443.  Approach to a tremor patient.  Sharma S(1), Pandey S(1). | 2016 |  |  |  | Tremor ist the most common movement disorder and is depicted in this study. Clinical features, diagnostic methods, rating scales for tremor severity and treatment ideas were carved out and described. | LR |
| Psychogenic tremor: a video guide to its distinguishing features. | 1. Tremor Other Hyperkinet Mov (N Y). 2014 Aug 27;4:253. doi: 10.7916/D8FJ2F0Q.  eCollection 2014.  Psychogenic tremor: a video guide to its distinguishing features.  Thenganatt MA(1), Jankovic J(1). | 2014 |  |  |  | This study provides video material and clinical characteristics to diagnose tremor and distinguish psychogenic tremor from other forms. Psychogenic tremor should not only be considered based on exclusion of organic causes, but should rely on features like abrupt onset, distractibility etc.. | MR |
| Psychogenic movement disorders in children and adolescents: an update. | 1. Eur J Pediatr. 2019 Apr;178(4):581-585. doi: 10.1007/s00431-019-03317-8. Epub  2019 Jan 11.  Psychogenic movement disorders in children and adolescents: an update.  Harris SR(1). | 2019 |  |  |  | Distribution of different psychogenic movement disorders, their underlying causes and characteristics were evaluated and described. Also most common treatment options are included and diagnostic tools although there are no standard methods for psychogenic movement disorders. | MR |
| Tremor: Sorting Through the Differential Diagnosis. | 1. Am Fam Physician. 2018 Feb 1;97(3):180-186.  Tremor: Sorting Through the Differential Diagnosis.  Crawford P(1), Zimmerman EE(1). | 2018 |  |  |  | Different forms of tremor like psychogenic tremor, essential tremor or parkinsonian tremor are hard to differentiate. Specific features of each form need to be defined, so that they can work as a guideline in diagnosing. Besides tremor characteristics single-photon emission computed tomography or transcranial ultrasonography can be used to distinguish tremor forms. | LR |
| Hurst revisited: Are symptoms and signs of functional motor and sensory disorders „dependent on idea“? | 1. J Neurol Sci. 2017 Oct 15;381:188-191. doi: 10.1016/j.jns.2017.08.3248. Epub 2017  Aug 24.  Hurst revisited: Are symptoms and signs of functional motor and sensory disorders  "dependent on idea"?  Stone J(1), Mutch J(2), Giannokous D(3), Hoeritzauer I(3), Carson A(2). | 2017 |  |  |  | For this experiment healthy non-medical adults were asked to pretend to suffer from motor and sensory symptoms. In different examinations their reactions and behavior were observed. The aim was to determine wether symptoms of patients with functional disorders depend on what the patient believes how symptoms express. | LR |
| Neurophysiologic studies of functional neurologic disorders. | 1. Handb Clin Neurol. 2016;139:61-71. doi: 10.1016/B978-0-12-801772-2.00006-0.  Neurophysiologic studies of functional neurologic disorders.  Hallett M(1). | 2016 |  |  |  | In this study underlying neurophysiological causes for functional neurologic disorders were examined. Brain regions were tested on proper or abnormal function. Speculations about the effect of inadequate function, especially lack of feedforward regulation, of the limbic system in functional disorders were made. | MR |
| Posttraumatic functional movement disorders. | 1. Handb Clin Neurol. 2016;139:499-507. doi: 10.1016/B978-0-12-801772-2.00041-2.  Posttraumatic functional movement disorders.  Ganos C(1), Edwards MJ(2), Bhatia KP(3). | 2016 |  |  |  | Besides severe traumatic injury to the nervous system, minor traumatic events may also lead to functional movement disorders. In these cases the connection between the injury and the onset often is contentious. This specific pathophysiology is discussed in this study as well as adequate management of psychogenic movement disorders. | MR |
| Functional (psychogenic) movement disorders - Clinical presentations. | 1. Parkinsonism Relat Disord. 2016 Jan;22 Suppl 1:S149-52. doi:  10.1016/j.parkreldis.2015.08.036. Epub 2015 Sep 3.  Functional (psychogenic) movement disorders - Clinical presentations.  Hallett M(1). | 2016 |  |  |  | Specific characteristics of different psychogenic movement disorders are given to distinguish for example psychogenic tremor, myoclonus or gait disorders. Also general features of functional movement abnormalities like sudden onset or underlying psychogenic causes are mentioned. | LR |
| Recognizing uncommon presentations of psychogenic (functional) movement disorders. | 1. Tremor Other Hyperkinet Mov (N Y). 2015 Jan 21;5:279. doi: 10.7916/D8VM4B13.  eCollection 2015.  Recognizing uncommon presentations of psychogenic (functional) movement  disorders.  Baizabal-Carvallo JF(1), Fekete R(2). | 2015 |  |  |  | Features like sudden onset, distractibility or entrainment help to distinguish the most common psychogenic movement disorders from one another. To diagnose uncommon psychogenic movement disorders further characteristics are needed. This study investigates clinical features in rare movement disorders. | LR |
| Psychogenic movement disorders. | 1. Neurol Clin. 2015 Feb;33(1):205-24. doi: 10.1016/j.ncl.2014.09.013.  Psychogenic movement disorders.  Thenganatt MA(1), Jankovic J(2). | 2015 |  |  |  | This study gives an overview over psychogenic movement disorders. Clinical features, diagnostic methods, treatment and the pathophysiology of different psychogenic movement disorders are discussed. | LR |
| Psychogenic movement disorders. | 1. Continuum (Minneap Minn). 2013 Oct;19(5 Movement Disorders):1383-96. doi:  10.1212/01.CON.0000436160.41071.79.  Psychogenic movement disorders.  Morgante F, Edwards MJ, Espay AJ. | 2013 |  |  |  | Psychogenic movement disorders were observed and described as to clinical features in diagnosis, pathophysiology and treatment. These abnormalities remain difficult to diagnose and to treat. Some patients benefit from cognitive strategies and physical rehabilitation, especially when the disorder was diagnoses short after onset. | LR |
| Trick or treat? Showing patients with functional (psychogenic) motor symptoms their physical signs. | 1. Neurology. 2012 Jul 17;79(3):282-4. doi: 10.1212/WNL.0b013e31825fdf63. Epub 2012  Jul 3.  Trick or treat? Showing patients with functional (psychogenic) motor symptoms  their physical signs.  Stone J(1), Edwards M. | 2012 |  |  |  | Showing patients with functional motor symptoms their physical signs to persuade them of the diagnosis is suggested in this study. Thereby patients should also be convinced of the potential reversibility of their symptoms. | LR |
| Pearls: hyperkinetic movement disorders. | 1. Semin Neurol. 2010 Feb;30(1):15-22. doi: 10.1055/s-0029-1245005. Epub 2010 Feb 1.  Pearls: hyperkinetic movement disorders.  Reich SG(1). | 2010 |  |  |  | In this study clinical characteristics for movement disorders are given. Presented are tremor, dystonia, chorea and ballismus as well as tics and myoclonus. Besides psychogenic and drug-induced movement disorders are mentioned. | LR |
| Psychogenic movement disorders: past developments, current status, and future directions. | 1. Mov Disord. 2011 May;26(6):1175-86. doi: 10.1002/mds.23571.  Psychogenic movement disorders: past developments, current status, and future  directions.  Lang AE(1), Voon V. | 2011 |  |  |  | This study of psychogenic movement disorders includes past developments in this subject and describes current diagnostic methods and clinical features as well as therapeutic strategies. The pathophysiology of such disorders is not yet fully understood and further progress must be made also regarding diagnostic and treatment. | LR |
| Physiology of psychogenic movement disorders. | 1. J Clin Neurosci. 2010 Aug;17(8):959-65. doi: 10.1016/j.jocn.2009.11.021. Epub  2010 May 20.  Physiology of psychogenic movement disorders. | 2010 |  |  |  | Physiology of psychogenic movement disorders still is mostly unknown. Some physiological test are available to diagnose and differentiate PMDs, especially tremor, but to differ psychogenic from organic dystonia remains a challenge. Also the mechanism of how a movement can be voluntarily produced but considered as involuntary is not yet identified. | LR |
